# Establishment of an Experimental Breast Cancer ZHENG Model and Curative Effect Evaluation of Zuo-Jin Wan

**DOI:** 10.1155/2013/324732

**Published:** 2013-11-24

**Authors:** Jia Du, Yang Sun, Xiu-Feng Wang, Yi-Yu Lu, Qian-Mei Zhou, Shi-Bing Su

**Affiliations:** Research Center for Traditional Chinese Medicine Complexity System, Shanghai University of Traditional Chinese Medicine, 1200 Cailun Road, Pudong, Shanghai 201203, China

## Abstract

Herbal formulas based on the traditional Chinese medicine (TCM) syndrome (ZHENG) have been used as alternative treatments for breast cancer. However, there is a lack of the experimental animal ZHENG model for the evaluation of the herbal formulas. In this study, we have established 4T1 mouse breast cancer with Liver Fire Invading Stomach Syndrome model (4T1 LFISS mice) and investigated the effects of the herbal formula, Zuo-Jin Wan (ZJW). Our results showed that 4T1 LFISS mice have the features of LFISS including irritability, loss of appetite, yellow urine, chow, and a tail hot. Compared to untreated 4T1 LFISS mice, ZJW significantly reduced tumor weight and volume (*P* < 0.05), although it was weaker than Cisplatin. However, ZJW significantly increased the body weight and food intake of 4T1 LFISS mice and decreased serum ALT, AST, Cr, and BUN levels and ZHENG score (*P* < 0.05), while Cisplatin reduced the food intake, and body weight and increased serum ALT, AST, Cr, and BUN levels in 4T1 LFISS mice. Our study has provided a mouse breast cancer ZHENG model and showed that ZJW suppresses tumor growth and improves LFISS and kidney and liver functions in the 4T1 LFISS mice.

## 1. Introduction

Breast cancer is the most common cancer among women; 1.38 million women were diagnosed with breast cancer in 2008. Its incidence rates vary considerably, with the highest rates in Europe and the lowest rates in Africa and Asia [1]. Its incidence rate in China is also increasing rapidly, and the number of cases increased by 38.5% from 2000 to 2005 [2]. However, there currently is a lack of clinically safe and effective drugs for prevention and treatment of breast cancer. In traditional Chinese medicine (TCM), some herbal formulas originating from natural products have been used as alternative treatments for breast cancer [3]. 

In order to experimentally evaluate the effectiveness and safety of treatment based-ZHENG (TCM syndrome) for cancer, an animal ZHENG model is necessary. However, there is a lack of an experimental animal ZHENG model in cancer research. Recently, Chen et al. [4] have established mouse xenograft pancreatic cancer models with dampness-heat, spleen-deficiency, and blood-stasis syndromes and found that they correlated with the treatment response of herbal medicine. An animal ZHENG models for breast cancer needs to be established for the evaluation of Chinese herbal formula. 

Zuo-Jin Wan (ZJW, also called Zuo-Jin pill) is a Chinese herbal formula. In the clinical practices of TCM, the efficacy of ZJW is based on Liver Fire Invading Stomach Syndrome (LFISS, a ZHENG), which is characterized by a choking sensation in the chest and irritability, loss of appetite, yellow urine, red tongue, and more in patients with cancer. Moreover, in recent studies, ZJW has been identified experimentally to have anticancer activity in recent studies in gastric cancer [5], liver cancer [6] and colorectal cancer [7], and multiple cancer cell lines, and it has suggested that the anti-cancer activities are due to induction of mitochondria-dependent apoptosis pathway [8]. However, whether ZJW has inhibitory activities against tumor growth without the side effects in breast cancer is unknown. 

In this study, we have established 4T1 mouse breast cancer with Liver Fire Invading Stomach Syndrome model (4T1 LFISS mice) and investigated the effects and safety of Zuo-Jin Wan (ZJW) compared to Cisplatin on 4T1 LFISS mice. Our results showed that ZJW inhibits the tumor growth and improves LFISS and kidney and liver functions in the 4T1 LFISS mice.

## 2. Materials and Methods

### 2.1. Cell Culture

The mouse 4T1 mammary tumor cell line was purchased from Shanghai Cell Bank, Chinese Academy of Sciences (Shanghai, China), and was cultured in DMEM medium (Gibco, San Francisco, CA, USA) supplemented with 10% heat-inactivated (56°C, 30 min) fetal calf serum (PAA, A-4061, Pasching, Austria), penicillin (100 U/mL), and streptomycin (100 *μ*g/mL). The cell culture was maintained at 37°C with 5% CO_2_ in a humidified atmosphere. 

### 2.2. 4T1 LFISS Mice

Female BALB/c mice (5 weeks old) were purchased from SLAC Laboratory Animal (Shanghai, China) and bred in the Laboratory Animal Center at Shanghai University of traditional Chinese medicine. The mice were housed in pathogen-free condition throughout the experimental duration and given free access to commercial rodent chow and water. 4T1 cells (3 × 10^6^, suspended in 100 *μ*L of PBS), were injected into mammary fat pads of female BALB/c mice. 

### 2.3. Preparation of Zuo-Jin Wan and Cisplatin

Zuo-Jin Wan is formulated by mixing the herbs, Rhizoma Coptidis and Fructus Euodiae as 6 : 1 ratio. The herbs were purchased from Shanghai Kangqiao Chinese Traditional Medicine Co., Ltd. (Shanghai, China). The aqueous extracts of these herbs was prepared and the quality control was performed as described previously [4]. The extract was stored at −20°C, and its preparations were standardized, regulated, and quality controlled according to the guidelines defined by the Chinese State Food and Drug Administration. Cisplatin injection was purchased from Nanjing Pharmaceutical Factory Co., Ltd. (Nanjing, China). 

### 2.4. Treatment Protocol

One day after tumor cell inoculation, the mice were randomly divided into four groups (*n* = 8 per group; normal, *n* = 6). ZJW-treated group (1.8 g/kg, once a day) was received by gavages, and Cisplatin-treated group (5 mg/kg, next day at a time), the positive control, was received by intraperitoneal injection as a positive control. Untreated groups were divided into a normal group and a model group. The model group was received with physiological saline as a sham control. The treatments lasted for 3 weeks. The mouse body weight, food intake, tumor weight, and volume were measured at different time points following tumor implantation. The tumor volume (*V*) was calculated by *V* = *a* (long diameter) ×  *b* (short diameter) 2 ÷ 2. 

### 2.5. Liver and Kidney Functions Tests

After obtaining blood samples by picked eyeballs of the mice, it was centrifuged at 3000 rpm for 10 minutes in order to separate and collect the serum. Serum alanine transaminase (ALT), aspartate transaminase (AST), serum creatinine (Cr), and blood urea nitrogen (BUN) were measured using the colorimeter testing kit (Kangcheng, Nanjing, China). According to the manufacturer's instructions, serum samples were measured at 510 nm, 510 nm, 510 nm, and 520 nm, respectively. 

### 2.6. Evaluation of ZHENG in Mice

The LFISS, a ZHENG, in 4T1 mice was diagnosed by its characteristic symptoms and signs, which include irritability, loss of appetite, yellow urine and claws, and a tail hot. It was given a semiquantitative evaluation as shown in Table 1, according to the established methodology and criteria for ZHENG animal models [9]. 

### 2.7. Efficacy Evaluation of ZHENG

The efficacy evaluation of ZHENG in mice was done according to the “Guideline for Clinical New Drug Research in Chinese Herbal Medicine” [10]. The standard of ZHENG outcome was scored as follows: ZHENG score as none: 0; light: 1 point; middle: 2 points; heavy: 3 points (Table 1). The calculation formula was as follows: the efficacy index of ZHENG (*N*) = [(before treatment score − after treatment score)/before treatment score] × 100%. The efficacy evaluation standard of ZHENG was the following: experimental cure: *N* ≥ 90%; excellent: *N* < 90% to 60%; effective: *N* ≤ 60% to >30%; invalid: *N* ≤ 30% [11]. 

### 2.8. Statistical Analyses

All data was expressed as means ± SD. Comparisons between groups were performed by Student's *t*-test and one-way analysis of variance (ANOVA). The level of significance was set at *P* < 0.05.

## 3. Results

### 3.1. ZJW Reduced Tumor Weight and Volume in 4T1 LFISS Mice

**Figure 1:**
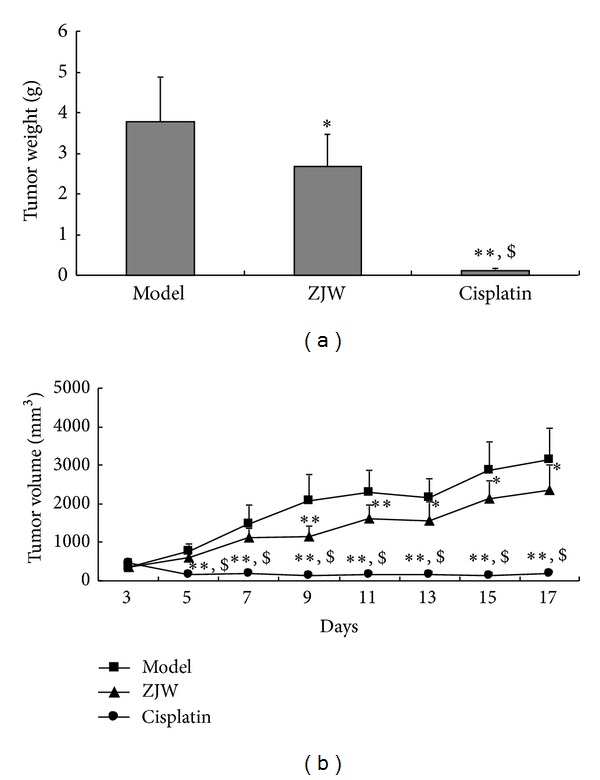
ZJW reduced tumor weight and volume in 4T1 LFISS mice. Tumor weights (a) and volumes (b) in 4T1 LFISS mice with ZJW and Cisplatin in treatments were measured, respectively. *P < 0.05, **P < 0.01, versus model group; ^$^ *P* < 0.05, versus ZJW-treated group.

To determine whether ZJW could suppress the tumor growth of breast cancer, we tested the ability of ZJW to reduce tumor weight and volume in 4T1 LFISS mice. The tumor weights were significantly reduced in the ZJW-treated group (*P* < 0.05) and the Cisplatin-treated group (*P* < 0.01) compared to the model group, respectively (Figure 1(a)). Moreover, the tumor volumes were significantly reduced in the ZJW-treated group on days 9 and 11 (*P* < 0.01) and on days 13, 15, and 17 (*P* < 0.05). It significantly reduced on 5 to 17 days (*P* < 0.01) in the Cisplatin-treated group compared to the model group (Figure 1(b)). There were significant differences between the ZJW-treated group and the Cisplatin-treated group (*P* < 0.05). Together, these results suggested that ZJW can suppress the tumor growth, although it was weaker than Cisplatin in 4T1 LFISS mice.

### 3.2. Effects of ZJW on Body Weight in 4T1 LFISS Mice

**Figure 2:**
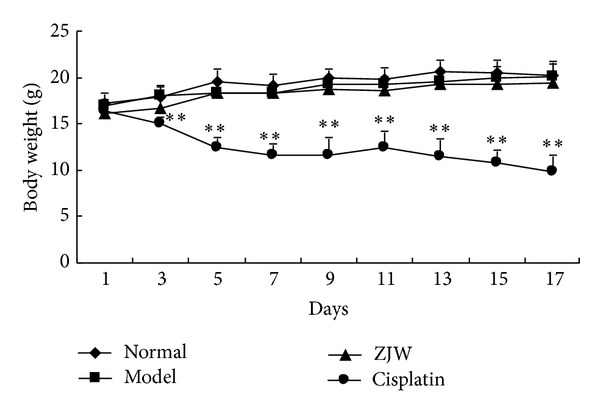
Effects of ZJW on body weight in 4T1 LFISS mice. Mice were treated by ZJW and Cisplatin. The body weights of 4T1 LFISS mice at different time points were measured. **P < 0.01, versus model group.

In order to detect whether ZJW has any side effects, we measured the body weight of 4T1 LFISS mice once each day. As shown in Figure 2(a), there were no significant differences of the body weight among normal group, model groups and ZJW-treated group (*P* > 0.05). However, starting from 3rd day, there were significant differences between Cisplatin-treated group (*P* < 0.01) and the other groups.

### 3.3. Effects of ZJW on ALT, AST, Cr, and BUN in 4T1 LFISS Mice

**Figure 3:**
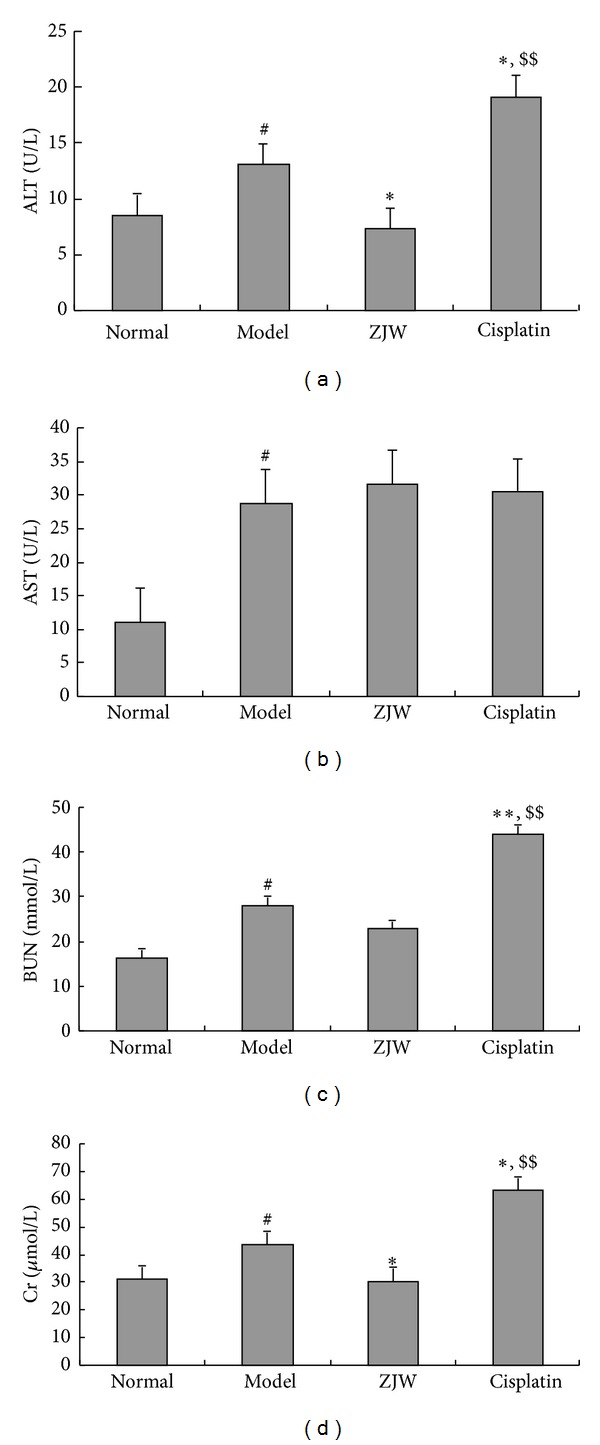
Effects of ZJW on kidney and liver functions in 4T1 LFISS mice. Mice were treated by ZJW and Cisplatin. (a) ALT, (b) AST, (c) BUN, and (d) Cr were measured using the colorimeter testing kit. *P < 0.01, versus model group; ^#^ *P* < 0.05, versus normal group; ^$$^ *P* < 0.01, versus ZJW-treated group.

We further tested whether ZJW has any effect on liver and kidney functions. The results showed that ALT (Figure 3(a)), AST (Figure 3(b)), Cr (Figure 3(c)), and BUN (Figure 3(d)) significantly increase in model group compared to normal group (*P* < 0.05). ALT (Figure 3(a)) and Cr (Figure 3(c)) were significantly decreased in ZJW-treated group compared to model group (*P* < 0.05), but there were no significant changes in the level of AST (Figure 3(b)) and BUN (Figure 3(d)) (*P* < 0.05). ALT (Figure 3(a)), AST (Figure 3(b)), Cr (Figure 3(c)), and BUN (Figure 3(d)) significantly increased in Cisplatin-treated group compared to model group (*P* < 0.05). Moreover, there were significant differences between ZJW-treated group and Cisplatin-treated group (*P* < 0.01). Together, these results suggested that ZJW has no side effects and improves liver and kidney functions while Cisplatin reduces body weight and increases ALT, AST, Cr, and BUN in 4T1 LFISS mice.

### 3.4. ZJW Increased Food Intake in 4T1 LFISS Mice

**Figure 4:**
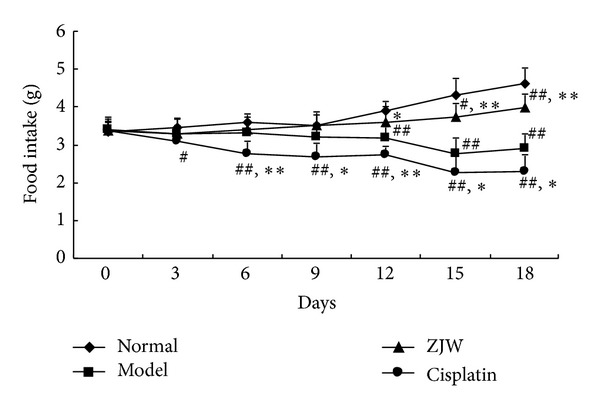
Effect of ZJW on food intake in 4T1 LFISS mice. The food intake in 4T1 LFISS mice with ZJW and Cisplatin treatments was measured. *P < 0.05, **P < 0.01, versus model group; ^#^ *P* < 0.05, ^##^ *P* < 0.01, versus normal group; ^$^ *P* < 0.05, ^$$^ *P* < 0.01, versus  ZJW-treated group.

In order to detect whether ZJW increased food intake, we measured the food intake of 4T1 LFISS mice once every 2 days. As shown in Figure 4, the food intake of 4T1 LFISS mice was significantly decreased in model group compared to normal group on days 12, 15, and 18, respectively (*P* < 0.05). Moreover, the food intake was significantly increased in ZJW-treated group compared to model group (*P* < 0.05 or *P* < 0.01) from the 15th day after treatment, but the food intake in Cisplatin-treated group was not only less than model group but was also significantly different from ZJW-treated group and normal group (*P* < 0.01). The results suggest that ZJW increased food intake while Cisplatin reduces food intake in 4T1 LFISS mice. 

### 3.5. ZJW Decreased ZHENG Score in 4T1 LFISS Mice

**Figure 5:**
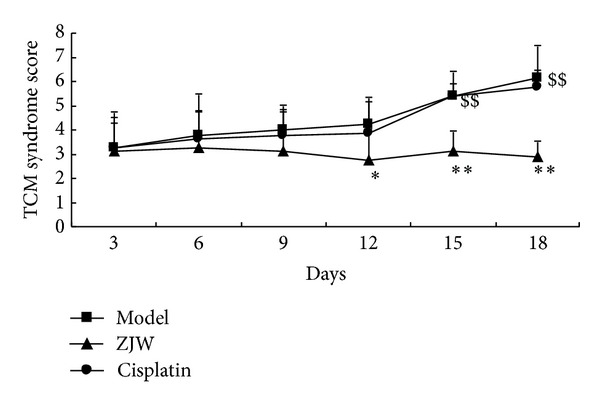
ZJW decreased ZHENG score in 4T1 LFISS Mice. The ZHENG scores in 4T1 LFISS mice with ZJW and Cisplatin treatments were measured. *P < 0.05, **P < 0.01, versus model group; ^$$^ *P* < 0.01, versus  ZJW-treated group.

To detect whether ZJW impacted the quality of life of the animals compared to Cisplatin treatment, we measured the ZHENG score of 4T1 LFISS mice once every 2 days. As shown in Figure 5, the ZHENG scores were significantly decreased in ZJW-treated group compared to model group (*P* < 0.05 or *P* < 0.01) from the 12th day after treatment, but Cisplatin-treated group was not significantly different from model group (*P* < 0.05), and the ZHENG scores were significantly decreased in Cisplatin-treated group compared to ZJW-treated group at day 15 and day 18, respectively (*P* < 0.01). The efficacy index of ZHENG is 53% in ZJW-treated group and 6.2% in Cisplatin-treated group, indicating that the ZJW treatment was effective and Cisplatin treatment was invalid. The results suggest that ZJW improves the quality of life of 4T1 LFISS mice through the decrease of ZHENG score while Cisplatin is unable to reduce ZHENG score in 4T1 LFISS mice.

## 4. Discussion and Conclusions

Treatment based on ZHENG differentiation, also called “Bian Zheng Shi Zhi”, is the comprehensive analysis of clinical information that is used to guide the choice of treatment with TCM herbal formulae [11, 12]. It is the main approach in the clinical practice to increase the effectiveness and safety of TCM treatment in the clinical practice. Following the revaluation of herbal formulas-based ZHENG, the development of new drugs and the discovery of the mechanisms all need an experimental animal model. Therefore, making the experimental animal ZHENG model as well as an animal disease model is important to breast cancer research. 

The animal ZHENG model is necessary for ZHENG research; however, there is a lack of the experimental animal ZHENG model in mice. Recently, Chai et al. have established a Deficiency of both Qi and Yin Syndrome (DQYS) model with the clinical features and one key pathological factor in mice [13]. Previous studies have also established mouse pancreatic cancer models for evaluation of the molecular mechanisms underlying ZHENG and tumor growth [14], and the administration of herbal medicine to the ZHENG model modified the tumor microenvironment [4]. In this study, a mouse breast cancer ZHENG model was established. It was 4T1 mouse mammary cancer ZHENG (LFISS) model, that is, 4T1 LFISS mice. The symptomatic features of 4T1 LFISS mice were irritability and loss of appetite, yellow urine, hot tails and/or claws, along with increase in tumor size (Figure 1), decrease in body weights (Figure 2), and liver and kidney functions disorders (Figure 3). Furthermore, it was found that the ZHENG of 4T1 LFISS mice responds to the ZJW treatment and does not responded to Cisplatin treatment, indicating that the established 4T1 mouse breast cancer LFISS model is useful for the evaluation of the efficacy of herbal formulas. 

Cisplatin is a common chemotherapy drug for breast cancer therapy, but its side effects such as nephrotoxicity [15], myelotoxicity [16], and neurotoxicity [17] limit its use. In this study, although Cisplatin treatment significantly suppressed tumor growth better than ZJW (Figure 1), it resulted in a decrease in body weight (Figure 2), liver and kidney functions disorders (Figure 3), and reduction in food intake (Figure 4). On the contrary, although the effect on inhibitory tumor is less than that of Cisplatin, ZJW increased body weight and food intake (Figures 2 and 4), improved liver and kidney functions (Figure 3), and improved the quality of life by reducing the ZHENG score (Figure 5) in 4T1 LFISS mice. It suggested that ZJW may be useful the as alternative treatment for breast cancer. Further research will investigate the effects of ZJW combined with Cisplatin.

Recurrently, the use of natural Chinese herbal medicine with antitumor effects is receiving to the treatment of breast cancer [18]. It is a holistic approach through multilevel, multitarget and multi-channel control, which focuses on reducing the side effects of chemotherapy, reversing drug resistance, and improving the quality of life and survival of patients. Therefore, these unique advantages have gradually made the TCM approach more promising in combating breast cancer [3, 19, 20]. Previous studies have shown that some herbal formulas have been used for the treatment of breast cancer including PC-SPESII extract [21] and Sangu decoction [20]. Although ZJW has been first found to have the antitumor effects on breast cancer in this study, the underlying mechanisms remain unknown.

ZJW consists of a combination of two Chinese herbs, Rhizoma Coptidis and Fructus Evodiae, in the ratio of 6 : 1 (w/w) [4, 22, 23], which contains high levels of active anti-neoplastic compounds such as berberine and evodiamine [24]. It has been reported that Rhizoma Coptidis and Fructus Evodiae synergistically inhibit s180 tumors in vivo [23], and the synergy of berberine and evodiamine enhances apoptosis of human hepatocellular carcinoma SMMC-7721 cells [24]. Moreover, it has been reported that evodiamine induces some cancer cell apoptosis, such as in human melanoma A375-S2 cells [25], human colorectal carcinoma COLO-205 cells [26], and human breast cancer MDA-MB-231 cells [25]. In addition, previous studies have shown that ZJW can inhibit the expression of inflammatory mediators [27] and modulate the monoaminergic neurotransmitter system [28] and catecholamine secretion [22]. These results indicated that the effects of ZJW may be involved in the induction of cancer cell apoptosis and the adjustment of the nerve, endocrine, and immune systems. 

In conclusion, the present study is aiming to establish a 4T1 mouse breast cancer ZHENG model and investigate the antitumor effects of the herbal formula, ZJW. Our study has established a mouse breast cancer ZHENG model and showed that ZJW suppresses tumor growth and improves the quality of life through reducing ZHENG score, improving the kidney and liver functions and without obvious side effects in the 4T1 LFISS mice. The results indicated that ZJW may be useful as alternative treatment for breast cancer. However, further research is needed to investigate the mechanisms of ZJW efficacy and safety. 

## Figures and Tables

**Table 1:** Semiquantitative evaluation of symptoms and signs.

Symptoms	Light (1 point)	Middle (2 points)	Heavy (3 points)
Irritability	Angry, irritable, and occasional mood swings	Angry, easily irritable, but can be controlled	Often be agitated and angry, and difficult to control
Loss of appetite	Poor appetite, amount eaten decreases by less than a third of original amount	Appetite reduction, amount eaten decreases by more than a third	No appetite, eat less than half
Yellow urine	Slightly yellow urine	Yellow urine	Deep yellow urine
Claws and tail hot	Claws and tail slightly hot	Claws and tail hot	Claws and tail very hot
